# Autoimmune Neutropenia Associated With HHV-6 Virus Infection: A Case Report

**DOI:** 10.3389/fimmu.2022.880016

**Published:** 2022-05-09

**Authors:** Kobi Faierstein, Noya Shilo, Asaf Levartovsky, Roy Raphael, Amir Givon, Nancy Agmon-Levin, Haim Mayan

**Affiliations:** ^1^ Department of Medicine E, The Chaim Sheba Medical Center, Ramat-Gan, Israel; ^2^ Sackler Faculty of Medicine, Tel Aviv University, Tel Aviv, Israel; ^3^ The Clinical Immunology, Angioedema and Allergy Unit, Center for Autoimmune Diseases, Sheba Medical Center, Ramat-Gan, Israel

**Keywords:** secondary autoimmune neutropenia (AIN), human herpesvirus 6 (HHV-6), granulocyte immunofluorescence test (GIFT), agranulocitosis, neutropenic fever

## Abstract

**Background:**

Autoimmune neutropenia (AIN) is divided into primary and secondary forms. The former is more prevalent in children and is usually a self-limiting disease. Secondary AIN is more common in adults and often occurs in the setting of another autoimmune disorder or secondary to infections, malignancies or medications. Several viral and bacterial pathogens were described to trigger AIN. Here we report a case of AIN in an adult woman associated with human herpesvirus-6 (HHV-6) infection.

**Case Presentation:**

We report a case of AIN in an adult woman associated with HHV-6 infection. The patient presented to the emergency department with fever and painful genital ulcers. Upon arrival, her laboratory workup demonstrated severe neutropenia and elevated inflammatory markers. She was hospitalized and underwent a thorough infectious, hematological, autoimmune and inflammatory workup. Malignancy was also excluded using an advanced whole body radiological scan. Serological tests confirmed the presence of both acute and chronic types of HHV-6 antibodies, at very high titers. Polymerase chain reaction demonstrated a numerous copies of the virus in the patient’s blood. Specific immunofluorescence test confirmed the diagnosis of autoimmune neutropenia.

**Conclusion:**

Secondary AIN is a rare disease that may affect all range of ages. The adult type is a challenging disorder that has different etiologies and may be triggered by a variable infectious pathogen. The finding of HHV-6 as a possible culprit pathogen may warrant physicians into widening the evaluation and include HHV-6 in the analysis.

## Introduction

Autoimmune neutropenia (AIN) is caused by increased destruction of peripheral neutrophils due to granulocyte-specific autoantibodies (1). The etiology is usually multifactorial and can be presented as primary or secondary in patients with other autoimmune diseases or an infectious trigger. Primary AIN is typically a pediatric disease of early infancy, manifests as a hematological abnormality, and is usually self-limiting. In contrast, secondary AIN is an adult-onset disease traditionally associated with other autoimmune diseases, infectious diseases, malignancies, or medications (2).

We report a case of a 30-year-old female who presented with fever and painful genital ulcers eventually diagnosed with autoimmune neutropenia associated with human herpesvirus type 6 (HHV-6) infection.

## Patient Description

A 30-year-old female presented with fever and painful genital lesions, beginning three weeks prior to her admission. At that time, she suffered from abscesses located on the fingers of her left hand, which were attributed to trauma. She began a course of antibiotic therapy with a first-generation cephalosporin and then switched to amoxicillin and clavulanate due to gastrointestinal adverse effects. Afterward, she reported painful gingivitis that was attributed to periodontitis. One infected tooth was extracted, and a prophylactic course of amoxicillin was prescribed. She consumed over-the-counter analgesics as non-steroidal anti-inflammatory drugs and dipyrone.

Her medical history included bipolar disorder, iron deficiency anemia, and an anal fissure treated conservatively. She also underwent bariatric surgery and a cholecystectomy ten years prior.

Physical examination was notable for several genital, labial and perineal, pus-secreting ulcers, fever of up to 38°C, and normal vital signs. Laboratory workup was remarkable for severe neutropenia of 0.13 K/microL neutrophils (range: 1.8-7.7 K/microL) with other than that unremarkable blood smear; acute kidney injury with creatinine 1.27 mg/dl (range: 0.51-0.95 mg/dl) and normal urinalysis. Markedly elevated inflammatory markers with C-reactive protein of 367 mg/l (range: 0-5 mg/l), and erythrocyte sedimentation rate were 84 mm/hour.

**Figure 1:**
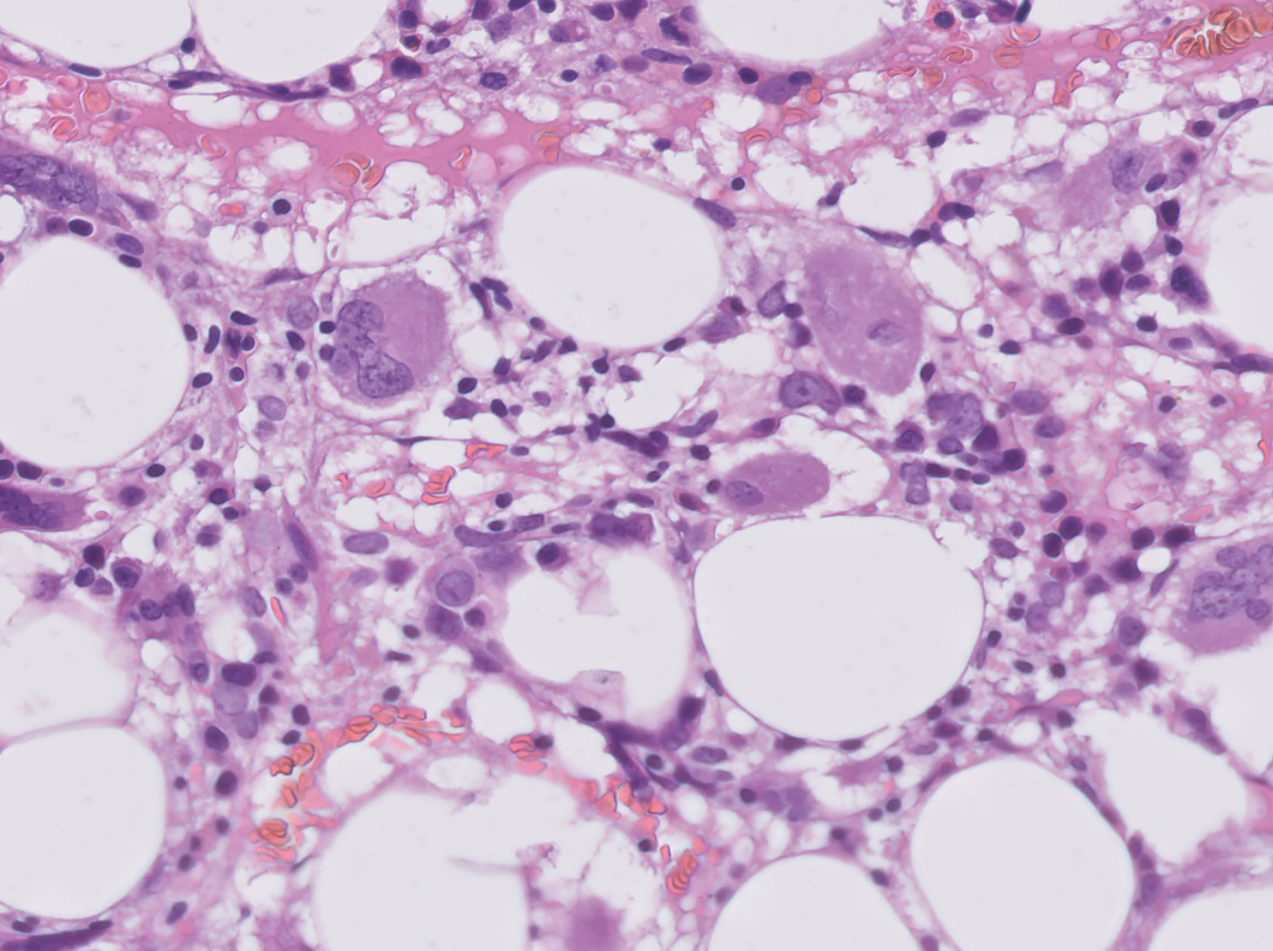
H&E section of the bone marrow showing vacuolization of the fat cells and absence of neutrophils.

She was primarily treated with intravascular hydration with fast recovery of kidney function, paracetamol, granulocyte colony-stimulating factor (G-CSF) and empiric broad-spectrum antibiotic. Further testing included cultures from genital ulcers swabs that were positive for Escherichia coli resistant to fluoroquinolones and Enterococcus faecium, leading to designated antibiotic treatment with vancomycin and aztreonam. A total body computed tomography scan did not demonstrate any source of infection and a positron emission tomography (PET) demonstrated high Fludeoxyglucose (FDG) uptake in the sternum and clavicles, mainly due to G-CSF therapy, but no evidence of other pathologies. A bone marrow biopsy demonstrated hypocellular bone marrow with agranulocytosis and evidence of stromal damage (**Figure 1**). This was followed by specific serologic analysis for autoantibodies against neutrophils, which included neutrophils of 8 different donors with known genotype. Our patient was found to have reactivity against neutrophils from all 8 different donors.

Daily treatment with G-CSF was not efficacious, and a trial of hydrocortisone therapy induced a flare of mania that required treatment discontinuation. The patient received a pre-treatment psychiatric consultation, treatment with olanzapine for two days and then treatments with 125 mg methylprednisolone for two consecutive days followed by oral glucocorticoids. After two days of methylprednisolone therapy, she was afebrile, and neutrophil count began to recover.

An additional workup reveled prolonged partial thromboplastin time (PTT), positive test for lupus anticoagulant and low complement of C3 and C4. A wide laboratory autoimmune panel including other anti-phospholipids antibodies was unremarkable. Avast rheumatological and immunological panel, including ANA, c-ANCA, p-ANCA, anti-dsDNA, anti-CCP, rheumatoid-factor, anti-RO, ani-LA and thyroid function was unremarkable. She underwent an extensive infectious workup that resulted in sterile blood and urine cultures. A test for HLA-B5 was negative excluding Behcet’s disease, although except the genital lesions, she had no other clinical signs or demographic background towards the disease. Viral serology and polymerase chain reaction (PCR) for cytomegalovirus (CMV), Ebstein-Barr virus (EBV), human immunodeficiency virus (HIV), herpes simplex (HSV), and varicella-zoster virus (VZV), were negative. Whereas a positive PCR with numerous copies of human herpesvirus 6 (HHV-6) was demonstrated in the patient’s blood, followed by positive serology for HHV-6 that was compatible with a very high IgG and IgM antibodies against the virus.

**Figure 2:**
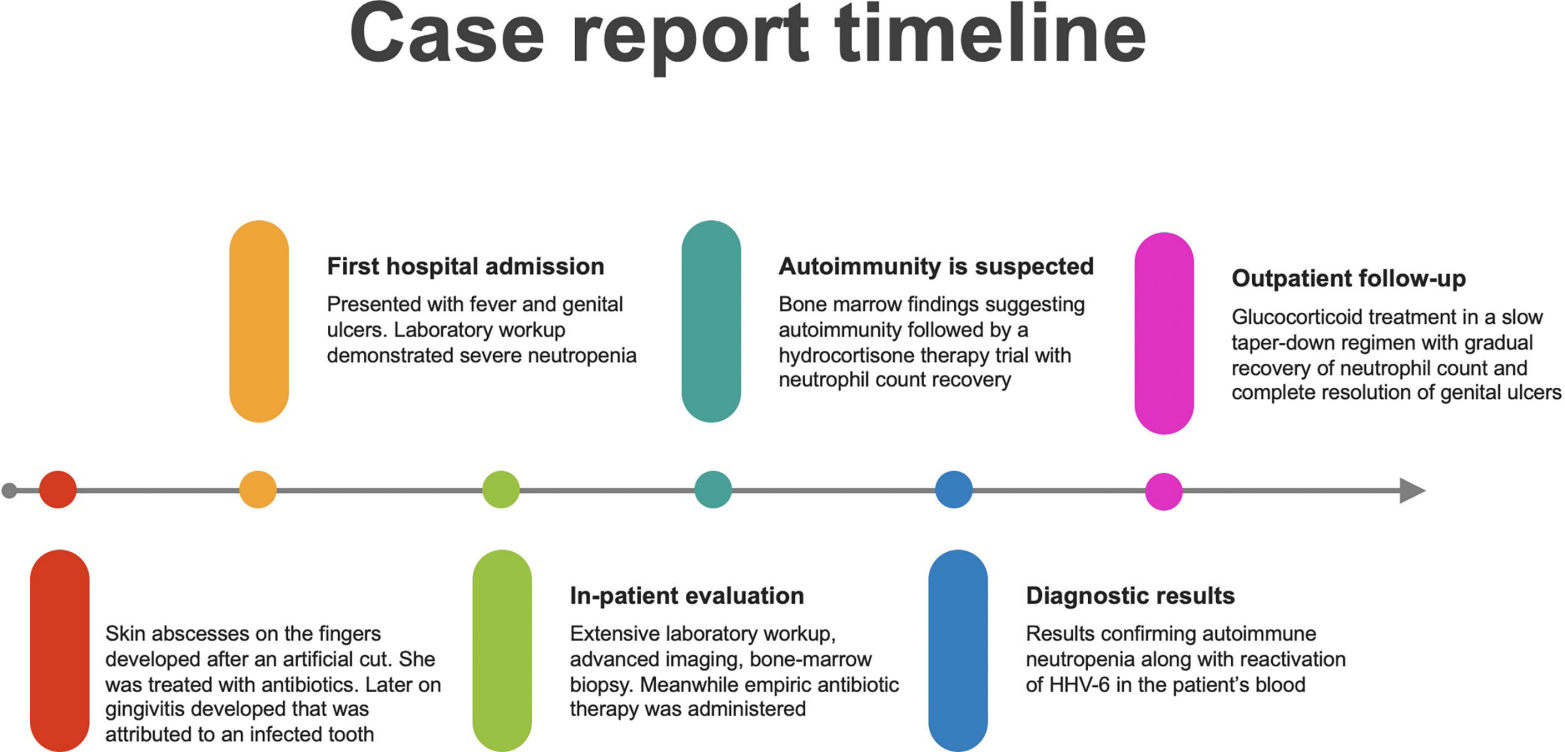
Timeline summarizing the process.

Eventually, a diagnosis of autoimmune neutropenia associated with HHV-6 infection was suggested and the patient was discharged with a very slow taper down regimen of glucocorticoids, and additional low dose aspirin. Her white blood cell count continued to recover until full normalization with no glucocorticoid therapy, and her genital ulcers gradually healed (**Figure 2**).

## Discussion

Several factors can trigger secondary autoimmune neutropenia. Infections are of the most common etiologies, including bacterial skin infections, upper respiratory tract, and otitis media. Viral infections can also be associated with secondary AIN, mainly human-immunodeficiency virus (HIV), hepatitis, and parvovirus B19. In this case report, we demonstrated the association of an HHV-6 infection with adult-onset AIN confirmed by the presence of anti-neutrophil antibodies. Adult-onset AIN is associated with underlying autoimmune diseases, infections or malignancies, and an extensive workup is frequently required to reach the etiological basis. In this case, our entire workup was negative; except for detection of HHV-6 is the patient’s blood and skin tissue.

There are no specific clinical manifestations of AIN, and the suspicion for this autoimmune disease is raised following a peripheral blood sample showing absolute neutrophil count of < 500 neutrophils/μL. There is no particular feature for AIN on bone marrow examination, however hypocellularity and stromal hyperplasia suggests an immune-mediated etiology, as was documented herein.

Establishing a diagnosis of AIN is a 2-step process. Initially, peripheral neutropenia, and secondly the demonstration of autoantibodies against neutrophils. For the diagnosis we used the granulocyte immunofluorescence test (GIFT), which is the most sensitive test (3). In the patient’s serum there were antibodies that acted against all 8 donors.

HHV-6 is a member of the beta-herpesvirus subgroup, typically affecting children or immunosuppressed individuals. It is mainly known for causing exanthem subitum, characterized by a prodrome of high fever lasting up to 5 days followed by a sudden onset of rash upon fever descent. Other clinical manifestations may include flu-like symptoms and even cause encephalitis. Our patient suffered from several painful genital and labial lesions.

There are two types of HHV-6, A and B, that cause distinct diseases (4). We assume that our patient was infected with HHV-6B type, given its higher prevalence in the population, however, in our case, we did not characterize the types. It has been shown that HHV-6 may lead to severe illnesses in immunocompromised patients, especially after allogenic hematopoietic stem-cell transplantation, including acute graft versus host disease, lower respiratory tract disease and encephalitis (5). There are some antiviral agents effective against the virus, however they were used, so far, only in the most severe cases (6). Given the young age of our patient, and the absence of significant past medical history, we decided not to treat her with antiviral medications that are pending further research.

Several autoimmune disorders have been suggested to be associated with HHV-6, albeit an underlying mechanism is still unclear. These include multiple sclerosis, myocarditis, idiopathic thrombocytopenia, and Sjogren’s syndrome (7). In the patient described herein, a high titer of lupus anticoagulant was demonstrated, yet clinical manifestations of antiphospholipid syndrome or systemic lupus were absent.

To the best of our knowledge, adult-onset AIN associated with HHV-6 infection has not been reported in the literature. Two reports described patients up to 12 months of age with HHV-6 infection manifested as transient erythroblastopenia and multiple brain abscesses (8, 9). Our patient presented with severe agranulocytosis and recent exposure to medications that could cause the insult. However, aborting all potential medications that were regularly used by the patient and treating with G-CSF did not result in recovery of her white cell count thus making a drug-induced neutropenia a less likely diagnosis.

A possible limitation of our case is that although we demonstrated a high viral load and positive serology during the acute illness, an entity of chromosomally-inherited HHV-6 (ci-HHV-6) was not excluded. About 1% of the population have an integrated viral DNA on the telomeres of each cell (10). This population usually have persistently elevated viral load in the blood. However, in our case, during her acute disease, on several tests her cycle time on PCR tests was low and both IgG and IgM antibodies were detected. Interestingly, on a follow-up meeting 4 weeks after discharge, still on a steroid taper-down regime, PCR for HHV-6 was negative and only IgG antibody was detected with a low titer. In case of a ci-HHV-6 we would not expect such a fast elimination of the virus. Given the fact that our workup revealed no other etiology, it may be plausible that the immunosuppression associated with AIN led to the reactivation of the virus.

To conclude, secondary AIN is an uncommon autoimmune disease that can be triggered by infectious agents as viral infections among patients of a wide range of ages. In particularly adult onset AIN is yet a challenging event, which requires a broad-spectrum assessment. The present finding of HHV-6 as a plausible viral trigger of AIN is reported here for the first time to the best of our knowledge, and is an addition to the scope of this disease. This may alert physicians treating patients with adult-onset AIN to expand evaluation and include HHV-6 analysis.

## Data Availability Statement

The raw data supporting the conclusions of this article will be made available by the authors, without undue reservation.

## Author Contributions

KF, NS, and AL wrote the original draft and the final version of the manuscript. All authors contributed to manuscript revision, read, and approved the submitted version.

## Conflict of Interest

The authors declare that the research was conducted in the absence of any commercial or financial relationships that could be construed as a potential conflict of interest.

## Publisher’s Note

All claims expressed in this article are solely those of the authors and do not necessarily represent those of their affiliated organizations, or those of the publisher, the editors and the reviewers. Any product that may be evaluated in this article, or claim that may be made by its manufacturer, is not guaranteed or endorsed by the publisher.
